# Phosphomevalonate kinase deficiency expands the genetic spectrum of systemic autoinflammatory diseases

**DOI:** 10.1016/j.jaci.2023.06.013

**Published:** 2023-10

**Authors:** Jakob Berner, Cheryl van de Wetering, Raul Jimenez Heredia, Christina Rashkova, Sacha Ferdinandusse, Janet Koster, Johannes G. Weiss, Alexandra Frohne, Sarah Giuliani, Hans R. Waterham, Irinka Castanon, Jürgen Brunner, Kaan Boztug

**Affiliations:** a St. Anna Children’s Cancer Research Institute (CCRI), Vienna, Austria; b St. Anna Children’s Hospital, Department of Pediatrics and Adolescent Medicine, Medical University of Vienna, Vienna, Austria; c Ludwig Boltzmann Institute for Rare and Undiagnosed Diseases, Vienna, Austria; d Department of Dermatology, Venereology and Allergology, Klinik Landstraße, Vienna, Austria; e CeMM Research Center for Molecular Medicine of the Austrian Academy of Sciences, Vienna, Austria; f Medical University of Vienna, Vienna, Austria; g Amsterdam UMC location University of Amsterdam, Department of Clinical Chemistry, Laboratory Genetic Metabolic Diseases, Amsterdam Gastroenterology Endocrinology Metabolism, Meibergdreef 9, Amsterdam, the Netherlands; h Department of Pediatrics, Medical University Innsbruck, Innsbruck, Austria; i Institute of Developmental Immunology, Medical University Innsbruck, Innsbruck, Austria; j Faculty of Medicine and Dentistry, Danube Private University, Krems, Austria

**Keywords:** Inborn errors of immunity, autoinflammation, genetics, PMVK, isoprenoid biosynthesis pathway

## Abstract

**Background:**

In the isoprenoid biosynthesis pathway, mevalonate is phosphorylated in 2 subsequent enzyme steps by MVK and PMVK to generate mevalonate pyrophosphate that is further metabolized to produce sterol and nonsterol isoprenoids. Biallelic pathogenic variants in *MVK* result in the autoinflammatory metabolic disorder MVK deficiency. So far, however, no patients with proven PMVK deficiency due to biallelic pathogenic variants in *PMVK* have been reported.

**Objectives:**

This study reports the first patient with functionally confirmed PMVK deficiency, including the clinical, biochemical, and immunological consequences of a homozygous missense variant in *PMVK*.

**Methods:**

The investigators performed whole-exome sequencing and functional studies in cells from a patient who, on clinical and immunological evaluation, was suspected of an autoinflammatory disease*.*

**Results:**

The investigators identified a homozygous *PMVK* p.Val131Ala (NM_006556.4: c.392T>C) missense variant in the index patient. Pathogenicity was supported by genetic algorithms and modeling analysis and confirmed in patient cells that revealed markedly reduced PMVK enzyme activity due to a virtually complete absence of PMVK protein. Clinically, the patient showed various similarities as well as distinct features compared to patients with MVK deficiency and responded well to therapeutic IL-1 inhibition.

**Conclusions:**

This study reported the first patient with proven PMVK deficiency due to a homozygous missense variant in *PMVK,* leading to an autoinflammatory disease. PMVK deficiency expands the genetic spectrum of systemic autoinflammatory diseases, characterized by recurrent fevers, arthritis, and cytopenia and thus should be included in the differential diagnosis and genetic testing for systemic autoinflammatory diseases.

## Introduction

Hereditary systemic autoinflammatory diseases (SAIDs) comprise a heterogeneous group of rare monogenic disorders as classified in subgroup VIIa of the current International Union of Immunological Societies phenotypic classification for human inborn errors of immunity.^1^^,^^2^ These disorders are characterized by recurrent and often unprovoked inflammatory episodes that include fever and varying degrees of autoinflammation. Most of them are caused by germline pathogenic variants in genes encoding for innate immune system components, including the inflammasome complex and the TNF receptor superfamily, but they have also been linked to enzyme deficiencies such as MKV deficiency (MKD).^2^^,^^3^

Biallelic pathogenic variants in *MVK* have also been found to result in an autoinflammatory disease, with a clinical spectrum ranging from periodic fever and autoinflammation to severe and early lethal clinical presentation as seen in patients with mevalonic aciduria.^4^, ^5^, ^6^ Patients with MKD usually excrete elevated levels of urinary mevalonic acid during disease flares.^6^ MVK is the first enzyme to follow the rate-limiting enzyme 3-hydroxy-3-methyl-glutaryl–coenzyme A reductase in the isoprenoid biosynthesis pathway. This pathway generates numerous sterol and nonsterol isoprenoids with important roles in multiple cellular processes, including cell growth, cell differentiation, signaling, and protein prenylation.^7^ MVK catalyzes the phosphorylation of mevalonate, a product of 3-hydroxy-3-methyl-glutaryl–coenzyme A reductase, to generate mevalonate-5-phosphate, which is subsequently phosphorylated by PMVK to generate mevalonate-5-diphosphate.^8^ Patients carrying pathogenic variants in *MVK* present on average every 2-12 weeks with multiple inflammatory symptoms such as recurrent fever with a typical duration of 3-7 days, diffuse maculopapular rash, polymorphous rashes, cervical lymphadenopathy, splenomegaly, chronic aphthae, polyarthritis, abdominal pain, vomiting, diarrhea, and elevated serum inflammatory markers, notably IL-1β.^9^ Reduced enzyme activity cause an accumulation of mevalonic acid and a decreased production of geranyl-geranyl-phosphate, leading to unchecked Toll-like receptor–induced inflammatory responses and constitutive pyrin inflammasome activation with IL-1 release via Rho A inactivation.^8^^,^^10^^,^^11^ In addition to MKD, which is inherited in an autosomal recessive fashion, localized autoinflammation in linear porokeratosis has been associated with pathogenic germline monoallelic *MVK* variants followed by a secondary somatic event within the same gene.^4^ Similarly, heterozygous variants in *PMVK* have been linked to linear porokeratosis.^12^

## Results and discussion

**Fig 1:**
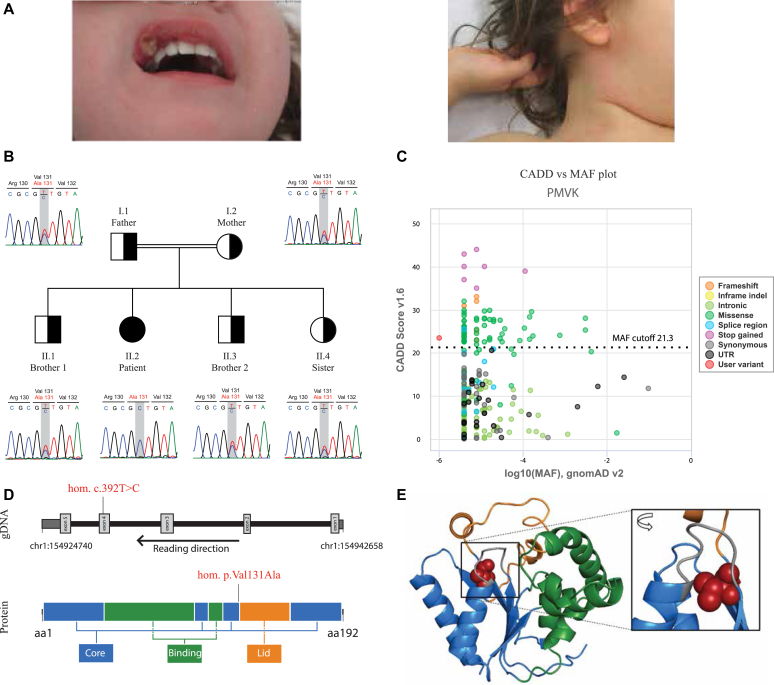
**A,** Aphthous stomatitis and maculopapular cervical rash in one febrile episode. **B,** Family pedigree and chromatograms of each family member with the p.Val131Ala variant highlighted. **C,** CADD score (y-axis) versus minor allele frequency (*MAF*) (x-axis) plot generated with PopViz (https://hgidsoft.rockefeller.edu/PopViz/) with the patient’s mutation highlighted in *red*. CADD score v1.6 and gnomAD v2.1.1. Mutation significance cutoff set with 95% CI**. D,***PMVK* genomic DNA (*gDNA*) and 2-dimensional protein structure with the location of the mutation highlighted. For the gDNA, the flat *dark gray boxes* flanking the figure represent untranslated regions (*UTRs*), the thick *light gray boxes* represent the exons, and the *black lines* represent the introns. For the protein, the different colors represent the different regions: core region in *blue*, acceptor substrate binding region in *green,* and Lid region in *orange*. **E,** Illustration of the 3-dimensional PMVK protein structure with the wild-type p.Val131 residue highlighted in *red* (*spheres*). The 3 protein domains are shown in *blue* (core region), *green* (acceptor substrate binding region), and *orange* (Lid region). The p-loop, a core element with a crucial role in phosphate binding, is shown in *gray*.

We report a now 5-year-old girl with recurring hyperinflammatory episodes. The patient descends from a family of Turkish origin and was born in Austria. Both parents and siblings (1 sister and 2 brothers) have been clinically unremarkable, and the family history was negative for inborn errors of immunity or SAIDs. The patient initially presented at 9 months of age with a hyperinflammatory episode including fever, arthritis, aphthous stomatitis, and maculopapular rash (Fig 1, *A* and Table I),^13^^,^^14^ but no trigger could be identified. Apart from this episode, the prior medical history was unremarkable. At the age of 2 years, the patient presented with a second episode of high fever (up to 40°C) and hepatomegaly, without splenomegaly or lymphadenopathy. Blood inflammatory parameters such as calprotectin (>25,000 μg/L [normal range <3,000 μg/L]), C-reactive protein (4.54 mg/dL [normal rage <0.5 mg/dL]), ferritin (1,442 μg/L [normal range 7-60 μg/L]), and erythrocyte sedimentation rate (130 mm/h [normal range <20 mm/h]) were elevated. Additionally, the patient showed severe hematological involvement with severe normochromic, microcytic anemia with hemoglobin (Hb) levels of 7.2 g/dL (normal range 9.5-14 g/dL). Hb electrophoresis showed a normal distribution of Hb fractions. Consistently, iron levels were normal. The patient also developed thrombocytopenia (25 × 10^3^/μL [normal range 150-450 × 10^3^/μL]) and granulocytopenia (0.3 × 10^3^/μL [normal range 2.5-7 × 10^3^/μL]). Due to the pancytopenia, a hematologic disease was considered. Bone marrow aspiration showed normal thrombo- and granulopoiesis with a strongly reduced erythropoiesis. Due to the differential diagnosis of inborn errors of immunity and SAIDs, a complete immunological workup was done. These results revealed normal kidney and liver function tests, normal immunophenotyping, and normal values for immunoglobulins (IgA, IgM, IgG, IgE) and IgG subclasses. An analysis of the complement system was unremarkable. Infectious diseases were excluded. Electrocardiography, echocardiography, electroencephalography, and the chest x-ray results were normal, and the abdominal sonography presented only the hepatomegaly. Supportive treatment with intravenous erythrocyte supplementation once and daily subcutaneous G-CSF (granulocyte colony-stimulating factor, 120 µg) injections were initiated for a total of 3 months, resulting in normalized neutrophil counts. Due to the suspected autoinflammatory disease, organic acid analysis of urine was performed during an episode of fever that revealed a significant elevated mevalonate to creatinine ratio of 11.8 μmol/mmol (normal range < 0.3), which is suggestive of MKD. Thus, genetic testing was performed. Initially, a targeted next-generation-sequencing–based protocol ruled out the presence of potentially pathogenic variants in the following genes: *MEFV, NLRP3, NOD2, MVK, NLRP12, TNFRSF1A, CARD14, IL10RA, PLCG2, SLC29A3, CECR1, IL10RB, PSMB8, TMEM173, IL1RN, IL36RN, PSTPIP1, TNFRSF11A, IL10, LPIN2, SH3BP2, TNFAIP3, NLRC4, HAX1, ELANE, G6PC3,* and *GFI1*. Next, whole-exome sequencing was performed and identified a rare homozygous variant in *PMVK* *(NM_006556.4: c.392T>C; p.Val131Ala).* The evaluation of all rare homozygous variants based on literature and criteria such as gene function, expression patterns, mouse model data, and deleteriousness-prediction scores including CADD (Combined Annotation Dependent Depletion) score, revealed *PMVK* p.Val131Ala as the most likely cause of disease. The variant segregated under the assumption of autosomal recessive inheritance and none of the healthy family members were homozygous for the variant (Fig 1, *B*). Homozygosity mapping based on whole-exome sequencing data indicated the existence of several homozygous regions, which points toward a distant degree of consanguinity between the parents of the index patient. Thus, additional homozygous variants with high prediction scores were observed, yet no pathogenic variants in *MVK* or other SAID genes could be identified. Further stringent filtering, segregation analysis, and literature research ruled out other heterozygous and homozygous variants. The identified *PMVK* variant was not found in the gnomAD (Genome Aggregation Database) (v3.1.2), or the GME (Greater Middle East Variome) database and bioinformatic prediction scores predicted a high likelihood of functional relevance (CADD v1.6 of 23.5) (Fig 1, *C* and Table II), suggesting that it may be disruptive to PMVK function. Structural analysis of the variant showed that the valine at p.131 is located in the β4 strand adjacent to the Lid domain. Through the buried position of its side chain, it has spatial proximity to the helix α_1_ linked to the p-loop, an element crucially involved in phosphate binding (Fig 1, *D* and *E*).^15^

**Table I:** Clinical table

Clinical characteristics	PMVK deficiency	MKD	
		Van der Hilst 2008^13^	Ter Haar 2016^14^
Patients, n	1	103	114
Age at onset (mo), median	9	6	6
Fever	Yes	100%	100%
Disease pattern			
Reoccurrence of attacks (wk)	2-12	NS	4
Duration of attacks (d)	3-7	NS	4
Recurrent	Yes	87%	NS
Continuous with exacerbations	No	8%	NS
Continuous	No	5%	NS
Mucocutaneous involvement			
Aphthous ulcers	Yes	48.5%	60%
Urticarial rash	Yes	68.9% (NS)	15%
Exudative pharyngitis	Yes	NS	28%
Maculopapular rash	No	68.9% (NS)	39%
Musculoskeletal involvement			
Arthralgia	Yes	83.5%	71%
Myalgia	No	NS	57%
Monoarthritis	Yes	55.3% (NS)	NS
Oligoarthritis	No	55.3% (NS)	NS
Polyarthritis	No	55.3% (NS)	57%
Eye involvement			
Periorbital edema	No	NS	NS
Uveitis	No	NS	2%
Conjunctivitis	No	NS	10%
Gastrointestinal involvement			
Abdominal pain	No	85.4%	88%
Diarrhea	No	71.6%	84%
Colitis	No	NS	NS
Hepatomegaly	Yes	21%	NS
Vomiting	No	70.9%	69%
Reticuloendothelial involvement			
Lymphadenopathy	No	87.4%	90%
Splenomegaly	No	32.4%	NS
Cardiovascular involvement			
Chest pain	No	NS	NS
Cutaneous vasculitis	No	NS	NS
Pulmonary involvement			
Respiratory infections	Yes	NS	NS
Neurological involvement			
Headache	No	62.7%	38%
Hematological involvement			
Anemia	Yes	NS	NS
Granulocytopenia	Yes	NS	NS
Thrombocytopenia	Yes	NS	NS
Acute phase parameters			
Leukocytes (10^3^/μL), median	8.3	15	NS
CRP (mg/dL), median (range)	5.8 (1.6-19)	16.3 (3.6-40.4)	NS
ESR (mm/h), median	152	76	NS

*CRP*, C-reactive protein; *ESR*, erythrocyte sedimentation rate; *NS*, not specified in the original paper.

**Table II:** Genetic characterization of the *PMVK* variant

Gene description	
HGNC_ID	HGNC:9141
CCDS SIZE (INCLUDING UTRS) (NM_006556.4)	1002
EXONS, N	5
LOEUF SCORE (GNOMAD V2.1.1)	1.395
UNIQUE PROTEIN-CODING VARIANTS (GNOMAD V2.1.1), N	97 missense, 8 pLoF

Variant description	
Genome Reference Consortium Human Build	B38
CHROMOSOME	Chr1
POSITION	154926404
NT_REF	A
NT_ALT	G
CDNA_CHANGE	NM_006556.4: c.392T>C
AA_CHANGE	p.Val131Ala
Gnomad_Af (Gnomad V3.1.2)	NA

***I****n silico* pathogenicity assessment	
PolyPhen2_HVAR_score (PolyPhen-2 v2.2.2)	0,992
PolyPhen_prediction	Probably damaging
SIFT_score (SIFT ENSEMBL 66)	0
SIFT_prediction	Deleterious
CADD_v1.6	23.5
REVEL (release May 3, 2021)	0.389
MutationTaster2 (update 2015)	0.999983
PROVEAN_score (version 1.1 ENSEMBL 66)	−3.86

*AA,* Aminoacid; *AF*, allel frequency; *ALT*, alternative allele; *CCDS*, Consensus coding sequence; *HGNC_ID*, HUGO Gene Nomenclature Committee identifier; *HVAR*, *HumVar* training set; *LOEUF*, Loss-of-function observed/expected upper bound fraction; *NA*, not available; *pLoF*, putative loss of function; *REF*, reference allele.

**Fig 2:**
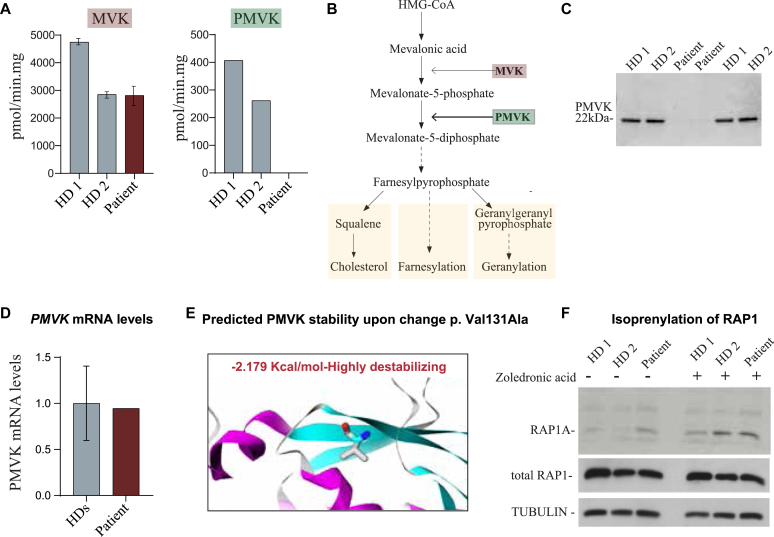
**A,** Enzyme activity measurements of MVK and PMVK activity in patient-derived lymphoblastoid cell lines in comparison to those of healthy donors (*HDs*). **B,** Simplified pathway of cholesterol biosynthesis indicating the defect in MKD and PMVK deficiency. **C,** Immunoblots of PMVK protein expression in patient-derived lymphoblastoid cell lines compared to those of HDs. **D,** Relative mRNA expression of *PMVK,* normalized to *PPIA* in patient-derived lymphoblastoid cell lines in comparison to those of 2 HDs. **E,** Predicted effect of the homozygous *PMVK* p.Val131Ala missense variant on PMVK protein stability using the mutation cutoff scanning matrix–stability method. **F,** Representative immunoblots of antibodies against unprenylated RAP1A and total RAP1 with and without zoledronic acid treatment. Tubulin was used as a loading control.

To investigate the consequences of the missense variant on PMVK function, we first measured PMVK and MVK enzymatic activity in B-lymphoblastoid cells derived from our patient and from healthy controls (Fig 2, *A*). While the MVK activity in patient cells was comparable to healthy controls, no PMVK enzymatic activity could be detected in patient cells (Fig 2, *A*), indicating that the missense variant abolished the ability of PMVK to transform mevalonate-5-phosphate to generate mevalonate-5-diphosphate. Assessment of PMVK protein levels using specific antibodies against PMVK showed a virtually complete absence of PMVK in patient-derived B-lymphoblastoid cells, explaining the deficient enzyme activity and demonstrating the pathogenic nature of the variant (Fig 2, *C*). This lack of protein expression is likely due to protein instability because the levels of *PMVK* mRNA were similar in patient- and healthy control–derived B-lymphoblastoid cells (Fig 2, *D*). Moreover, *in silico* thermodynamic protein stability prediction analysis on single-point mutation^9^ suggested that the PMVK p.Val131Ala variant had a strong effect on protein instability (Fig 2, *E*), indicating that protein instability is the most likely cause for the complete absence of PMVK protein expression.

It has been suggested that the inflammatory phenotype in patients with MKD is due to defects in the synthesis of geranylgeranyl-diphosphate, which affects the prenylation and function of downstream small guanosine triphosphatases, including RAP1A and RHOA.^10^ This subsequently leads to inflammasome activation resulting in an increased secretion of inflammatory cytokines such as IL-1β and IL-18.^10^^,^^16^ Based on this, we hypothesized that the inflammatory phenotype in the patient with PMVK could also result from defects in protein prenylation. To address this, we analyzed the levels of unprenylated RAP1A and found a clear accumulation of unprenylated RAP1A in our patient compared to healthy donors (Fig 2, *F*). This suggests that, similar to patients with MKD, defective protein prenylation is a possible mechanism to explain the enhanced inflammatory responses and the trigger of inflammatory flares observed in our patient with PMVK (Fig 2, *F*).

**Fig 3:**
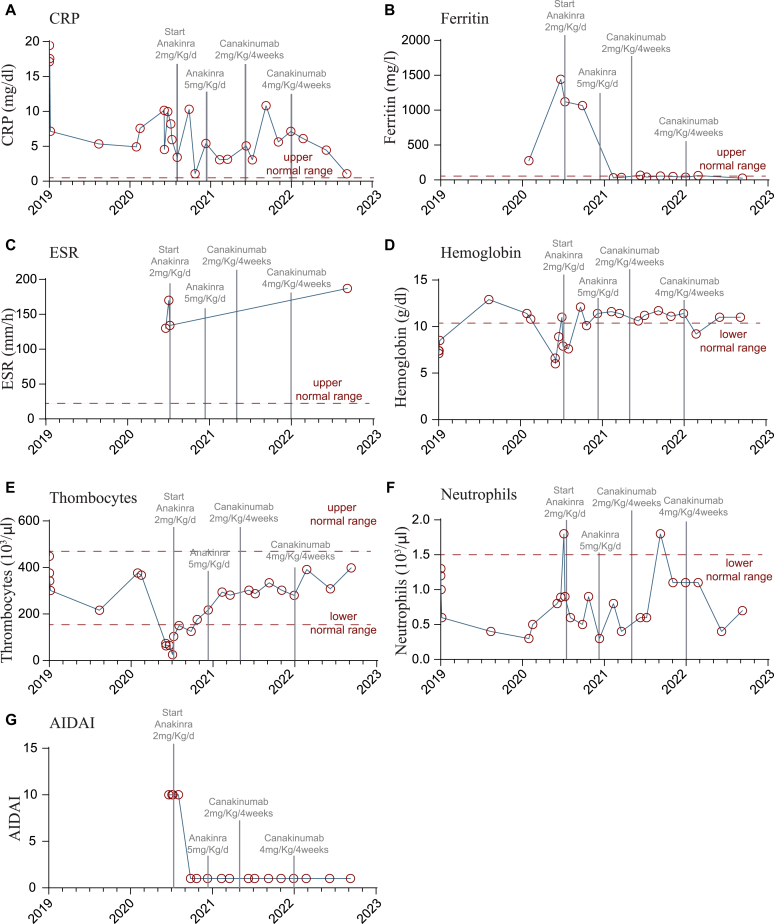
Clinical laboratory parameters over time. *Light gray lines* indicating the start and change of IL-1 inhibitory therapy. **A,** Serum levels of C-reactive protein (*CRP*). **B,** Serum levels of ferritin. **C,** Erythrocyte sedimentation rate (*ESR*). **D,** Hb levels. **E,** Thrombocyte count. **F,** Absolute neutrophil count. **G**, AIDA score.

It has been shown that many patients with MKD respond well to anti–IL-1 therapies, therefore we initiated an empiric treatment with an IL-1 inhibitor (anakinra initially dosed at 2 mg/kg body weight per day, followed by dosage increase to 5 mg/kg) (Fig 3).^3^ During the treatment, the severity of fever episodes decreased significantly and inflammatory parameters such as C-reactive protein and ferritin rapidly decreased (Fig 3, *A* and *B*). The erythrocyte sedimentation rate initially declined but remained increased over time (Fig 3, *C*). In addition, Hb levels and thrombocyte counts increased on treatment (Fig 3, *D* and *E*), while absolute neutrophil counts remained low (Fig 3, *F*). The overall positive response to IL-1 inhibition is represented by a clear reduction of the AIDA (Autoinflammatory Disease Activity) score (Fig 3, *G*). After 8 months, the anti–IL-1 treatment was switched from anakinra to canakinumab (initially dosed 2 mg/kg body weight, later 4 mg/kg) because canakinumab only has to be applied every 4 weeks and showed a similarly effective control of disease manifestations as anakinra (Fig 3).

To compare with patients with MKD, we assessed IgD levels, as well as serum cytokines including soluble IL-2 receptor, IL-6, IL-8, IL-10, and TNF-α in our patient, but were only able to measure these after treatment with IL-1 inhibitors and during clinical remission. Under these conditions, we did not observe an increase in serum cytokines under clinical remission of the patient (soluble IL-2 receptor: 6.6 ng/mL [0.9-11.5 ng/mL], IL-6: 2.9 pg/mL [0.0-10.1 pg/mL], IL-8: 6 pg/mL [0-28 pg/mL], and TNF-α: 18 pg/mL [0-32pg/mL]), except for IL-10, which was mildly elevated (4.5 pg/mL [0.0-3.5 pg/mL]). Similarly, levels of serum IgD were found to be within the upper limit (97 U/mL [normal range 0-100 U/mL]).

Clinically, our patient showed overlap with patients with MKD (Table I). However, our patient differs from patients with MKD in 2 aspects. First, she did not develop gastrointestinal symptoms such as abdominal pain or diarrhea, which is reported in up to 88% in patients with MKD.^13^^,^^14^ Second, she presented with pancytopenia, which is very uncommon in patients with milder MKD.^17^, ^18^, ^19^ IL-1 inhibition in our patient led to normalization of pancytopenia.

Notably, shortly prior to submission of this manuscript, a report described a patient with compound heterozygous variants in *PMVK*.^20^ The reported 6-year-old male patient presented with MKD-like inflammatory symptoms and compound heterozygous missense *PMVK* variants, one classified as likely pathogenic and the other as variant of unknown significance according to the American College of Medical Genetics classification. The consequences of the variants on PMVK activity or protein were not characterized in further detail, such as by investigation of protein expression and/or enzymatic activity, and thus causality was not fully demonstrated.

In summary, we here provide functional evidence that pathogenic variants in *PMVK* can cause a SAID that is clinically reminiscent of the autoinflammatory phenotype of MKD. Thus, *PMVK* should be included in diagnostic testing for SAIDs, and genetic testing is indicated in patients with an autoinflammatory disease, elevated urine mevalonate, and no pathogenic variants in *MVK.* Identification of additional patients may enable a delineation of the full phenotypic spectrum of this novel type of SAID. Furthermore, treatment approaches for PMVK deficiency can be aligned with MKD including nonsteroidal anti-inflammatory drugs, IL-1 and IL-6 inhibition, and anti–TNF-α therapies.^3^

**Clinical implications**

PMVK deficiency causes systemic hyperinflammation. PMVK deficiency should be included in genetic testing for SAIDs. IL-1 inhibition provides a valuable therapeutic option.

## Disclosure statement

This project has received funding from the 10.13039/501100000781European Research Council under the European Union's Horizon 2020 Research and Innovation Programme (ERC Consolidator grant agreement 820074 to K.B.). C.vdW. has been supported by a postdoctoral fellowship from the P.T. Engelhorn Foundation.

Disclosure of potential conflict of interest: The authors declare that they have no relevant conflicts of interest.

## Study oversight

The studies including immunologic diagnostic procedures and genetic analyses were performed in accordance with guidelines of good clinical practice, the current version of the Declaration of Helsinki, with written informed consent from the patient’s legal representatives, and with approval from the relevant institutional review boards.

## Whole-exome sequencing

For whole-exome sequencing of the patient, a TrueSeq Rapid Exome kit (Illumina, San Diego, Calif) as well as the Illumina HiSeq3000 system and the cBot (Illumina) cluster generation instruments were used as previously described^E1^^,^^E2^ with minor changes. Briefly, reads were aligned to the human genome version 19 by means of the Burrows-Wheeler Aligner.^E3^ Variant Effect Predictor was used for annotating single nucleotide variants and insertions/deletions lists. The obtained list was then filtered according to the presence of variants with a minor allele frequency >0.01 in the 1000 Genomes Project, gnomAD, and dbSNP (Single Nucleotide Polymorphism Database) build 149. After further filtering steps for nonsense, missense, and splice-site variants using an in-house developed software, an internal database was used to filter for recurrent variants. Moreover, variants are prioritized using tools, such as SIFT, PolyPhen-2, and CADD score,^E4^ that predict the deleteriousness of a present variant.

## Sanger sequencing

Sanger sequencing was used to validate the *PMVK* variant NM_006556.4: c.392T>C, p.(Val131Ala) in exon 4 from whole-exome sequencing in the affected patient and her family members. This was done by designing specific primers for the variant found: Fw 5′- CACTGAAGCTCCCAGCAGAA -3′, Rv 5′- CCCACCTCCCCTCACTTCTA -3′.

## Lymphoblastoid b-cell culture

Lymphoblastoid cell lines were generated from PBMCs as previously described.^E1^ Briefly, PBMCs were isolated using Ficoll (MilliporeSigma, Burlington, Mass) density centrifugation and incubated with EBV supernatant. The next day, 1 μg/mL cyclosporin A was added to the cells. In the following weeks, the cells were maintained in complete RPMI medium (Gibco, Thermo Fisher Scientific, Waltham, Mass) supplemented with 10% FCS, 1% L-glutamine, and 1% PenStrep (all from Invitrogen, Thermo Fisher Scientific).

## Detection of unprenylated RAP1A

For the detection of unprenylated RAP1A and total RAP1pr, lymphoblastoid B-cell lines of our patient and 2 healthy controls were cultivated in RPMI medium, containing 5% sterol-depleted FCS (MilliporeSigma), 1% L-glutamine, and 1% PenStrep. Cells were untreated or treated overnight with 20 μmol/L zoledronic acid (#SML0223; MilliporeSigma). Subsequently, the cells were washed and lysed in 50 mmol/L HEPES, 15 mmol/L NaCl, and 2 mmol/L MgCl_2_ and 1% protease inhibitor cocktail (#P8340, MilliporeSigma). Next, cells were sonicated using Covaris (Wolburn, Mass) sonicator (6 × 16 mm) tubes and used for immunoblotting using anti-RAP1A (diluted 1:250; #373968; Santa Cruz Biotechnology, Dallas, Tex) RAP1 (diluted 1:250; #398755; Santa Cruz Biotechnology), and α-Tubulin (diluted 1:5000; #18251; Abcam, Boston, Mass) antibodies.

## Immunoblotting

Lymphoblastoid cells were lysed in a PBS solution containing 0.1% SDS and Complete Mini protease inhibitors (Roche, Mannheim, Germany). Equal amounts of protein were separated by nuPAGE (Invitrogen, Thermo Fisher Scientific) and transferred onto nitrocellulose. Immunoblot analysis was performed with affinity purified rabbit polyclonal antibodies against PMVK (diluted 1:250).^E5^ Detection was done after incubation with secondary antibodies IRDye 800CW goat anti-rabbit (diluted 1:10,000) on the Odyssey Infrared Imaging System (LI-COR Biosciences, Lincoln, Neb).

## Enzyme activity measurements

MVK and PMVK activities in lymphoblast homogenates were measured radiochemically using ^14^C-labeled mevalonate prepared from a mixture of RS-mevalolactone (#M4667; MilliporeSigma) and R,S-[^2-14^C] mevalonolactone (American Radiolabeled Chemichals, St Louis, Mo) under alkaline conditions (100 mmol/L NaOH for 30 minutes at 37°C). Incubations with a final volume of 150 μL were performed at 37°C for 30 minutes and had the following composition: 100 mmol/L potassium phosphate (pH 7.0), 6 mmol/L MgCl_2_, 4 mmol/L ATP, 167 μmol/L [^2-14^C]-mevalonate (≈0.2 μCi), and 30 to 50 μg lymphoblast homogenate (prepared in phosphate buffered saline). Reactions were terminated by adding 50 μL 20% (vol/vol) formic acid. After 10 minutes at 37°C, the samples were put on top of a DEAE-Sephadex A25 column (1 mL; MilliporeSigma) equilibrated with H_2_O. The columns were washed with 10 mL 5 mmol/L HCl to elute the unreacted mevalonate. Separation between the different products was achieved by elution with 35 mmol/L HCl (5-phosphomevalonate) and 200 mmol/L HCl (5-pyrophosphomevalonate and other downstream phosphorylated products).

## RT-qPCR

Total RNA was extracted from lymphoblastoid B cells of 2 healthy controls and the patient using the RNeasy mini kit (Qiagen, Valencia, Calif) following the manufacturer’s instructions. Then 1 μg of RNA was reverse transcribed to cDNA using the miScript Reverse Transcription kit (Bio-Rad Laboratories, Hercules, Calif) and SYBR Green Universal Taq Mastermix (Bio-Rad Laboratories) to assess mRNA expression of *PMVK*. The data were analyzed using the ΔΔCt method and the expression values were normalized to the house keeping gene *PPIA*. Quantitative PCR was performed using the following primer pairs: *PMVK* (Fw 5′ TTTTGCAGGAAGATTGTGGA 3′, Rv 5′ CTCCGTGTGTCACTCACCA 3′), *PPIA* (Fw: 5′ ACGCCACCGCCGAGGAAAAC 3′; Rv: 5′ TGCAAACAGCTCAAAGGAGACGC 3′).
